# Finding the Optimal Pose of 2D LLT Sensors to Improve Object Pose Estimation

**DOI:** 10.3390/s22041536

**Published:** 2022-02-16

**Authors:** Dominik Heczko, Petr Oščádal, Tomáš Kot, Adam Boleslavský, Václav Krys, Jan Bém, Ivan Virgala, Zdenko Bobovský

**Affiliations:** 1 Department of Robotics, Faculty of Mechanical Engineering, VSB—Technical University of Ostrava, 70800 Ostrava, Czech Republic; petr.oscadal@vsb.cz (P.O.); tomas.kot@vsb.cz (T.K.); adam.boleslavsky@vsb.cz (A.B.); vaclav.krys@vsb.cz (V.K.); jan.bem.st@vsb.cz (J.B.); 2 Department of Industrial Automation and Mechatronics, Faculty of Mechanical Engineering, Technical University of Kosice, 04200 Kosice, Slovakia; ivan.virgala@tuke.sk

**Keywords:** orientation estimation, laser scanning, LLT sensor, virtual scanning, optimal configuration, optimal pose, ICP algorithm, pose estimation

## Abstract

In this paper, we examine a method for improving pose estimation by correctly positioning the sensors relative to the scanned object. Three objects made of different materials and using different manufacturing technologies were selected for the experiment. To collect input data for orientation estimation, a simulation environment was created where each object was scanned at different poses. A simulation model of the laser line triangulation sensor was created for scanning, and the optical surface properties of the scanned objects were set to simulate real scanning conditions. The simulation was verified on a real system using the UR10e robot to rotate and move the object. The presented results show that the simulation matches the real measurements and that the appropriate placement of the sensors has improved the orientation estimation.

## 1. Introduction

In industrial manufacturing, accuracy and precision requirements in assembly and manipulation of objects keep increasing. For a precise assembly performed by an industrial robot, it is important to define the object’s picking point and orientation so that the robot can accurately grasp the object and perform the assembly. Traditionally, this is done using process pallets, jigs, and other equipment. However, these jigs tend to be expensive and must be unique for each object. There are systems that perform assembly based on automatic guidance for large objects [1], where the robot end-effector must have a sensory subsystem to ensure sufficient precision. In [2], the authors deal with assembly of small components, where they use one camera in the end-effector to guide the robot to grasp objects, two cameras in the alignment phase of the object before assembly, followed by feedback from the force sensor during assembly; however, the object was correctly oriented for robot grasping. In the study [3], the authors achieve significant assembly accuracy with an industrial robot to hundredths of a degree by on-line movement compensation. However, they used a very expensive laser tracker sensor and were not dealing with grasping of objects.

Precise assembly is especially difficult in applications where objects in a box or bin are oriented chaotically. This bin-picking problem is solved in different ways, such as finding the position to grasp an object using a 3D camera [4] or object detection and pose estimation using a depth map [5]. An important part of bin-picking is image processing and recognition; nowadays, the use of neural networks has shown great promise in this task [6,7]. Although the pose estimation of the neural network is becoming more accurate, there is still an error in the estimation of orientation [8]. These solutions are sufficient for pick-and-place applications [9], such as unloading parts from a box onto a conveyor or other device. However, in order to achieve an exact grasping position for future manipulation, the part must be aligned with various jigs and fixtures. The additional alignment of parts for grasping by the robot is time-consuming and it would be more beneficial to eliminate this intermediate step. This can be achieved by performing the assembly process directly after grasping the object via bin-picking, thus eliminating the need for additional equipment to align objects and saving space by excluding the conveyor that is often used to transport objects to the next station.

For precise manipulation of objects grasped by bin-picking, it is necessary to determine their position and orientation in the robot’s gripper. This can be achieved by various methods, such as pose estimation from a 2D image (mainly RGB image) using neural networks [10,11]. RANdom SAmple Consensus (RANSAC) method is often used to fit 3D shape primitives and roughly estimates the pose of a carried object analyzing a 3D image of a depth camera or laser sensor data—point cloud [12]. To refine pose estimation, the Iterative Closest Point (ICP) algorithm [13] is used, which is very stable and robust and has been greatly improved in recent years [14].

Data collection can be provided by various sensors, generally divided into two groups: contact and non-contact. For contact sensors, tactile sensors are used which are placed on the fingers of the end-effector [15]. Eventually, the robot that holds the grasped object is driven into a metrology system [16] where the measurement will be performed. Tactile sensors are very accurate and insensitive to the optical properties of the scanned surfaces. However, obtaining a 3D shape of a complex part using a tactile sensor is time-consuming and contact with the measured part could lead to surface degradation. Therefore, contactless sensors are the preferred choice in today’s industry. Time-of-flight [17], triangulation (1D, 2D, and 3D) [18], or classic RGB cameras image analysis [19] are the most commonly used methods. When using non-contact sensors, the measured object cannot be damaged in any way and the measurement is faster; sensors can sense several thousand points within milliseconds [20]. However, contactless sensors are sensitive to optical properties of the scanned objects, especially shiny, very smooth, and transparent surfaces are problematic, and scanning data may be distorted or even absent. For example, in laser line triangulation (LLT) scanning, the laser beam may reflect off shiny surfaces outside the sensor and the laser line is not detected. Sensorics for detecting an object in the gripper is either placed directly in the end-effector [21] or the object is scanned by a sensor placed in the workstation [22].

In this work, we focused on the appropriate placement of LLT sensors with respect to a scanned object to provide relevant input data for pose estimation using RANSAC and ICP algorithms and thus improve the accuracy of orientation estimation, because the correct input data are essential for pose estimation methods. Finding the optimal sensor pose would be very time-consuming in a real environment, so a simulation environment was created to speed up the process. The optical properties of scanned objects were implemented in the simulation environment to simulate the real conditions during scanning. Three objects were selected for the experiment, each made with a different technology and material. The simulation results were verified on a real system. Besides, the simulation environment could be used to test the LLT sensor and its behavior on different materials during scanning before purchasing the actual sensor.

## 2. Methodology

As mentioned earlier, the data acquired by LLT sensors could be affected by the geometrical and optical properties of the sensed surfaces. Diffuse light reflection is important for LLT sensor scanning, which is the scattering of reflected light into the surroundings and thus into the charge-coupled device (CCD), which detects the laser line on the scanned surface. In previous research, we have investigated the effect of the angle of incidence (AoI) of the laser beam on the intensity of reflected light to ensure sufficient reflected light to the CCD sensor [23]. The research was performed for materials commonly used in the industry (plastics, metals, and others). These data were implemented in a simulation to simulate real conditions during scanning with an LLT sensor (specifically the Keyence LJ-X8080 sensor [24]) in a virtual environment.

**Figure 1:**
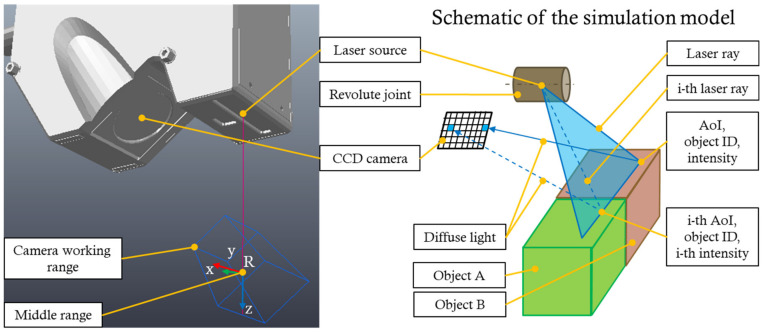
Simulation model of the LLT sensor LJ-X8080. Middle range is denoted as R and represents the middle range of the sensor—the coordinates at this point are X, Z (0, 0).

The simulation model was created in the CoppeliaSim environment [25]. The sensor simulation model returns the same data format as the actual sensor (3200 values for the height—Z-coordinate of each point); if a point was not detected, its value is −9999.9999 (empty point). The X-coordinate must be calculated according to the set resolution of the CCD camera. The simulation uses the available elements modified by custom scripts. Figure 1 describes the simulation model of the LLT sensor, where the vision sensor element was used to simulate the CCD camera; the proximity laser sensor (PLS) element was used to simulate the laser source.

The proximity laser sensor is controlled using the script described in Algorithm 1. The PLS is positioned using a revolute joint (as can be seen in Figure 1). If the laser ray (beam) hits the scanned object at a given position, it will detect its ID and surface properties (if there were multiple objects in the simulation, each may have different properties). The angle of incidence of the laser ray (beam) relative to the surface normal is calculated allowing to evaluate the intensity of the reflected laser ray. The obtained data are sent to the CCD camera script for further processing.
**Algorithm 1.** Evaluation of the reflected laser ray (beam) intensity.**for** i = 0 to m  // m[1:3200] represents the position of PLS     **set_laser_position** (i)     **if** sensing       **get_material_param** (ID)  // The ID represents the ID of the object on which the laser beam is incident       **compute_angle_of_incidence** (i)       **compute_intensity** (AoI)      **end if** **end for** **return** intensity // intensity represents an array of 3200 intensity values

The CCD camera is used to detect the laser line on the surface of the scanned object. The control script of the CCD camera is described by Algorithm 2. First, the intensity data from the PLS script are requested. Then the image is processed: the pixel columns are sequentially checked and the laser color is searched. If the color has been detected and the intensity of a given pixel is greater than the minimal detectable intensity, the height of the point (Z-coordinate) is written into the array. If the colour has not been detected or the intensity is below the threshold, the value −9999.9999 (blank point) is written into the array of coordinate values.
**Algorithm 2.** Laser line detection by CCD vision sensor.**get_intensity** () // intensity represent array of intensity of each point **for** i = 0 to m  // m[1:3200] represents a column in the camera image    **for** j = 0 to n   // n[1:2230] represents a row in the camera image      **if** RGB is detected         **if** intensity > min_intensity_value             **get_coordiante_of_point** (i)          **end if**      **end if**   **end for** **end for** **return** coordinates // coordinates represents an array of 3200 

The ICP algorithm available from the Open3D [26] and PCL [27] libraries was used to calculate the orientation of the object. Both libraries are open source and have been in continuous development. The input data given to the ICP algorithm are two point clouds—floating and fixed. The floating point cloud is fitted (registered) to the fixed point cloud, which is a reference point cloud. In our case, point clouds of three differently placed sensors relative to the scanned object and their configurations are used as the input (floating point clouds) to the algorithm.

**Figure 2:**
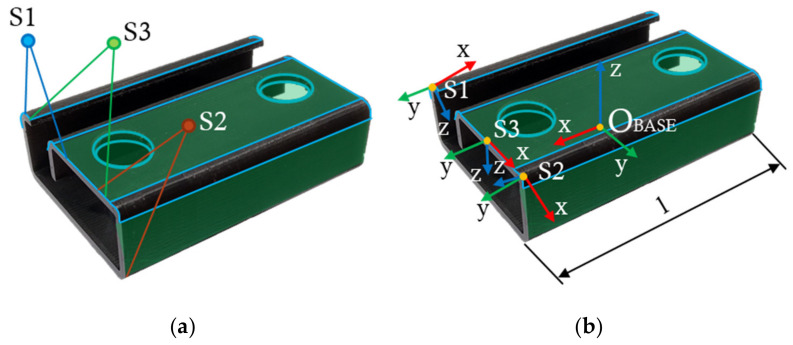
Methodology of LLT sensor placement (S1 is sensor no. 1, S2 is sensor no. 2, S3 is sensor no. 3): (**a**) the measurement ranges of sensors S1, S2, and S3; (**b**) coordinate systems S1, S2, and S3 represent the reference coordinate systems of LLT sensors, O_BASE_ is the object coordinate system; l is the scanning length.

To accurately register point clouds, it is essential to capture reference features (geometric primitives) in the floating point cloud such as demanded surfaces, edges, curves, cylinders, etc. An example of sensor placement is shown in Figure 2a, where the ranges of three differently placed LLT sensors are marked. Sensor 1 (S1) senses the left surface, part of the upper surface, the inner surface, and the roundness; Sensor 2 (S2) senses the upper surface and the holes; Sensor 3 (S3) senses the right surface, part of the upper surface, and the roundness. Figure 2b shows the positioned reference coordinate systems of the LLT sensors relative to the base coordinate system of the scanned object (O_BASE_). Length of the component is denoted as l and in this case, it is the same as the scanning length. Details of the experiment and its verification are described in Section 3.

## 3. Selection of Sensor Configurations and Their Verification

**Figure 3:**
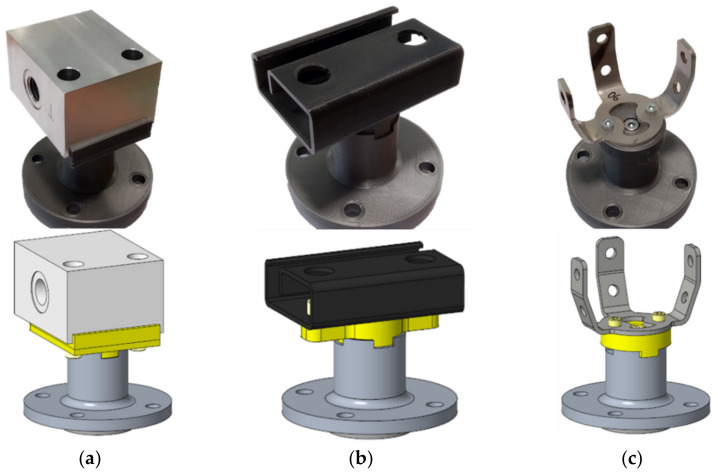
Objects for the experiment and their CAD models. All objects are connected to a flange that attaches to the UR10e for real measurements: (**a**) Part A and its CAD model (aluminum, roughness Ra1.6); (**b**) Part B and its CAD model (black PLA); (**c**) Part C and its CAD model (steel).

Three different objects were chosen for the experiment, which can be seen in Figure 3a,b. Each object had a different shape and material and was made with a different technology. Figure 3a shows Part A, which was made of aluminum alloy by milling, the surface roughness is Ra1.6; the dimensions of this part are 54 × 45 × 32 mm (length × width × height). Figure 3b shows Object B, which was made of plastic using additive manufacturing; the dimensions of this component are 60 × 41 × 17.6 mm. Furthermore, Figure 3c shows Part C, which was made by bending a steel cutout; the dimensions of this component are 42.5 × 55 × 32.5 mm. The material properties of Part C were not measured; however, the surface of this object is very smooth. Therefore, the material for the simulation was estimated as an aluminum alloy with the surface roughness of Ra0.8. For Objects A and B, the scanning length was identical to the length of the objects, while for Object C the scanning length was 12 mm, which is the width of the bent elements.

In the simulation environment, the reflection properties (the dependence of the AoI of the laser beam on the reflected intensity) were set according to [23] for each scanned object. Sensors were placed relative to the object coordinate system manually based on the user’s decision so that they captured as many reference features as possible during the scanning process (similar to Figure 2b). For each part, three sensor poses were predefined, from which the optimal ones were found (with the lowest pose estimation error). However, multiple sensor poses could be predefined for each object as well. Each component had a different shape, so the sensors were placed individually for each object. An example of the placement of sensors around Part B shows Equations (1)–(3), which represent a rigid transformation between the coordinate system of the object and the sensors coordinate system.

(1) $$M_{O_{Base}\to S1}=\left(\begin{array}{cccc} 0 & 1 & 0 & 0.03\\ 0.7071 & 0 & 0.7071 & -0.023\\ 0.7071 & 0 & -0.7071 & 0.015\\ 0 & 0 & 0 & 1 \end{array}\right)$$

(2) $$M_{O_{Base}\to S2}=\left(\begin{array}{cccc} 0 & 1 & 0 & 0.03\\ 1 & 0 & 0 & 0\\ 0 & 0 & -1 & 0.175\\ 0 & 0 & 0 & 1 \end{array}\right)$$

(3) $$M_{O_{Base}\to S3}=\left(\begin{array}{cccc} 0 & 1 & 0 & 0.03\\ 0.7071 & 0 & -0.7071 & 0.014\\ -0.7071 & 0 & -0.7071 & 0.015\\ 0 & 0 & 0 & 1 \end{array}\right)$$

Then, a series of measurements was taken for each object as described in Algorithm 3. First, the orientation of the object was set and then the scanning was performed. The orientation was set for all possible rotation combinations using RPY angles (Roll, Pitch, and Yaw):

- Rotation around a separate axis (Roll only, Pitch only, Yaw only);
- Rotation around two axes (Roll and Pitch only, Pitch and Yaw only, Yaw and Roll only);
- Rotation around all axes at once (Roll, Pitch, and Yaw).

**Algorithm 3.** Data collection algorithm.**set_object_orientation**(Roll, Pitch, Yaw) **for** i = 0 to m // m represents scanning steps (according to scanning length)    **set_object_position**(i)    **get_sensor_data**()    **transform_data**() **end for** **save_data**()

**Figure 4:**
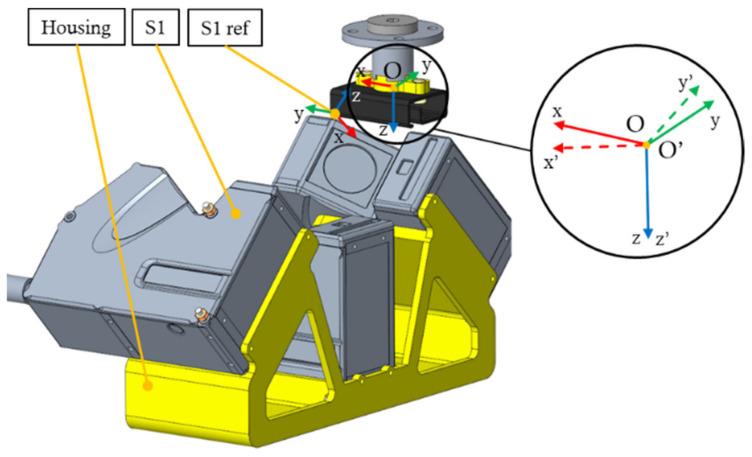
Scanning of Object B. The figure detail shows the coordinate systems O (the object is not oriented) and O’ (the object is oriented at a yaw angle of −2°). The scanning direction is always in the x-axis direction of the O coordinate system.

The Roll, Pitch, and Yaw angles were set from −2° to +2° in increments of 0.4°. This boundary was set based on the accuracy of the pose estimation of the bin-picking systems. The scanning length was set according to the length of the part or the width of the scanned feature, and the scanning step was set to 0.5 mm. During scanning, the object always moved forward in the x-axis direction of the O coordinate system (fixed coordinate system of the scanned object), as shown in Figure 4 in detail.

To create a 3D point cloud, the sequentially collected sensor data with respect to the sensor reference coordinate systems (marked as S1 ref in Figure 4 for sensor 1) must be expressed with respect to the object coordinate system (marked as O in Figure 4), which is achieved by the transformation in (4):

(4) $$P_{iO}=T_{Sjref\to O}^{-1}\cdot P_{i_{Sjref}}$$

where $P_{i_{Sjref}}$ is the *i*-th point expressed in the *j*-th sensor coordinate system, $T_{Sjref\to O}^{-1}$ is the transformation matrix from the *j*-th sensor coordinate system to the object coordinate system, and $P_{iO}$ is the *i*-th point expressed in the object coordinate system.

### 3.1. Selection of Sensor Configuration in Virtual Environment

From the dataset obtained from the simulations, the orientation was calculated as described in Algorithm 4. The point clouds of each sensor were entered into the calculation according to the configuration. After pre-processing, the point clouds were registered. From the obtained transformation matrix, the RPY angles (Roll, Pitch, and Yaw) and the deviation from the actual (set) orientation were calculated.
**Algorithm 4.** Point cloud registration.**for** i = 0 to m // m [1:7] represents the sensor configuration number   **for** j = 0 to n // n [1:11] represents the point clouds of each rotation (from −2° to +2°)      **merge_point_clouds**()  // according to sensor configuration      **process_point_cloud**()  // downsample, remove outliers      **point_cloud_registration**()      **get_rpy_angles**()      **compute_error**()      **save_data**()   **end for** **end for**

Based on the results, the best sensor configurations were selected—the sensors that had the smallest deviation in the calculation. The median of total error and the error range (75th percentile) were used to select the configurations. The orientation deviations are shown in the following figures using a boxplot, where the horizontal line in the boxplot represents the median error and the white dot represents the mean error.

**Figure 5:**
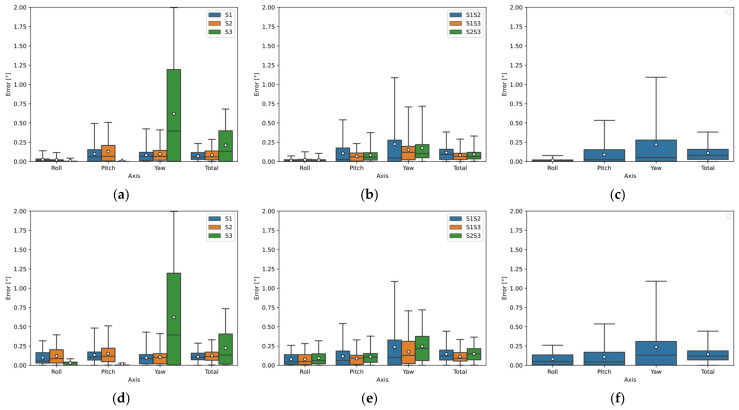
Object A orientation detection error by simulation using Open3D and PCL library: (**a**) sensors separately (Open3D); (**b**) two sensors configuration (Open3D); (**c**) three sensors configuration (Open3D); (**d**) sensors separately (PCL); (**e**) two sensors configuration (PCL); (**f**) three sensors configuration (PCL).

Figure 5a–c show the deviations of the calculated orientation of all sensor configurations for Object A using the Open3D library; Figure 5d–f show the deviations of the calculated orientation using the PCL library. The orientation estimation results were similar; however, the Open3D library performed better. Sensor S1 showed the best results using one sensor. Sensors S1 and S3 showed the best results using two sensors, where Sensor S1 data was used to calculate Roll and Yaw; Sensor S3 data were input to calculate Pitch. The best results using the three sensors were merged data of sensors S1, S2, and S3 to calculate Roll; for the calculation of Pitch data of Sensor S3; and for the calculation of Yaw data of Sensor S1.

**Figure 6:**
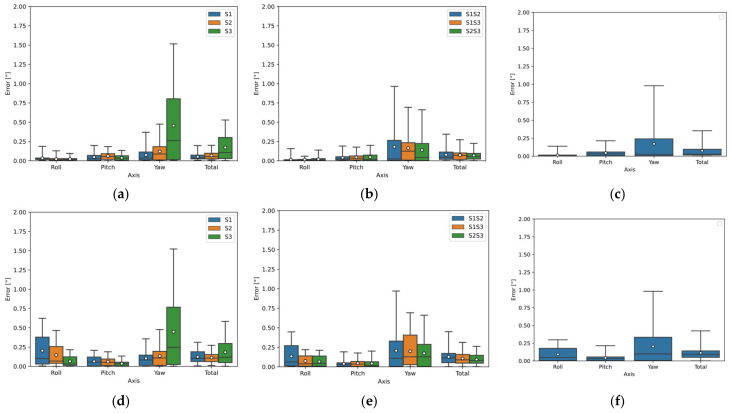
Object B orientation detection error by simulation using Open3D and PCL library: (**a**) sensors separately (Open3D); (**b**) two sensors configuration (Open3D); (**c**) three sensors configuration (Open3D); (**d**) sensors separately (PCL); (**e**) two sensors configuration (PCL); (**f**) three sensors configuration (PCL).

Figure 6a–c show the deviations of the calculated orientation of all sensor configurations for Object B using the Open3D library and Figure 6d–f using the PCL library. According to the orientation estimation results, the Open3D library achieved more accurate results. Sensor S1 showed the best results using one sensor. Sensors 1 and 2 showed the best results using two sensors, where the merged data from Sensors S1 and S2 were used to calculate Roll and Pitch; Sensor S1 was input for the calculation of Yaw. The best results using all three sensors were the merged data of Sensors S1, S2, and S3 for the calculation of Roll; the merged data of Sensors S2 and S3 for the calculation of Pitch; and the data of Sensor S1 for the calculation of Yaw.

**Figure 7:**
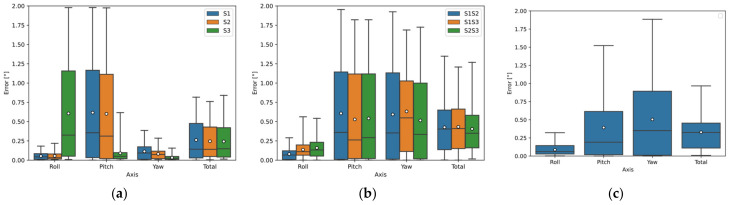

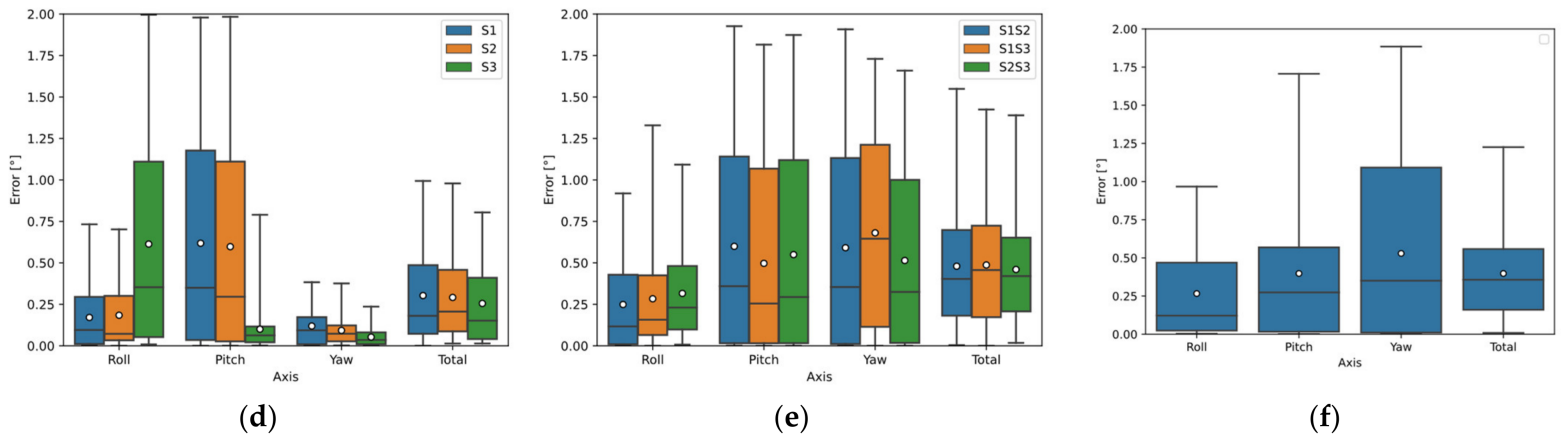
Object C orientation detection error by simulation using Open3D and PCL library: (**a**) sensors separately (Open3D); (**b**) two sensors configuration (Open3D); (**c**) three sensors configuration (Open3D); (**d**) sensors separately (PCL); (**e**) two sensors configuration (PCL); (**f**) three sensors configuration (PCL).

Figure 7a–c show the deviations of the calculated orientation of all sensor configurations for Object C using the Open3D library and Figure 7d–f using the PCL library. Again, the Open3D library provided more accurate orientation estimation results. The S2 sensor showed the best results using a single sensor. Sensors 2 and 3 showed the best results using two sensors, where Sensor S1 data was input for Roll calculation; Sensor S3 data was input for Pitch and Yaw calculation. The results using all three sensors were worse than using two sensors.

Based on the simulation results, the sensor configurations with the smallest deviation of the calculated orientation were selected. Configurations using one, two, and three sensors were used. The chosen configurations were verified on a real system. For Object C, only configurations using one and two sensors were selected because the three-sensor configuration performed worse than the two-sensor configuration. The orientation estimation results for both libraries were similar, but in all cases the results using the PCL library were worse. Therefore, in the next section, only the Open3D library was used for the calculation. The configurations were selected according to the results using the Open3D library, but the same configurations would have been selected using the PCL library (with worse results).

### 3.2. Measurement on a Real System

**Figure 8:**
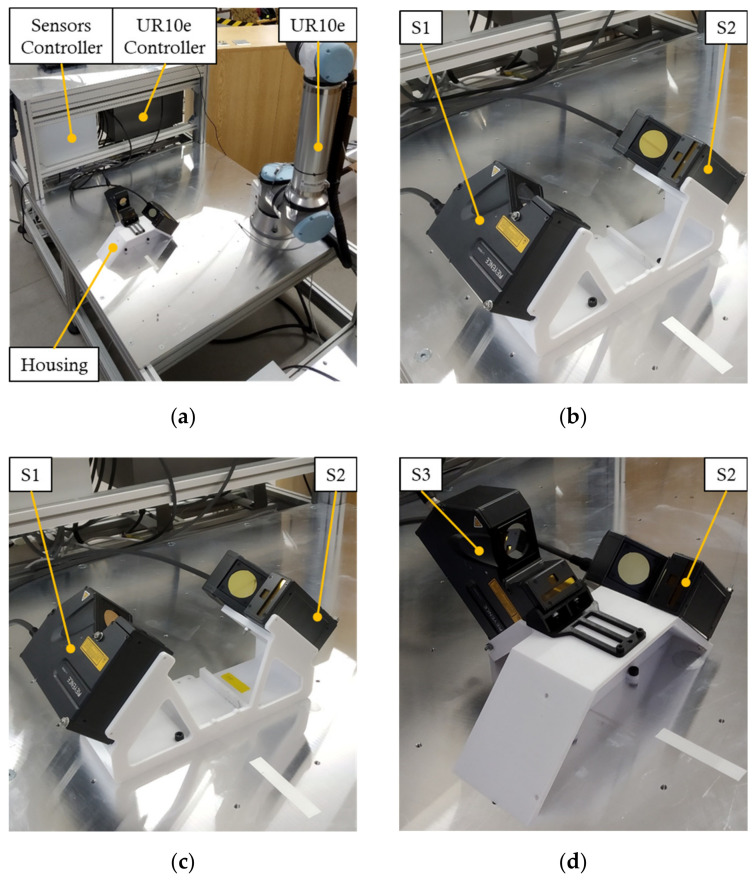
Experimental workplace and sensorics housings for all samples: (**a**) experimental workplace; (**b**) housing of Object A; (**c**) housing of object B; (**d**) housing of object C.

A workstation with the UR10e robot was used to verify the simulation results. The experimental workstation can be seen in Figure 8a. The UR10e robot was used to rotate the objects and to perform linear motion of the objects for scanning. During the measurement process, the robot heats up, which causes positioning inaccuracies, so we compensated the robot positioning accuracy according to [28].

Since we only had two LLT sensors, when measuring a three-sensor configuration, the data from two sensors were collected, then the data from the missing sensor were measured separately. For each object, a housing was created to hold the sensors in a defined position, as shown in Figure 8b–d. The housings were made by an additive manufacturing technique using PLA material, which is commonly used and has very good mechanical properties [29].

The housings were attached to an aluminum matrix with threaded holes, so their position relative to the robot base was known. There are always inaccuracies during manufacture and assembly, so the base of the housings with the robot base was additionally measured by [30] using 3D placement of Aruco tags [31] to obtain a static transformation matrix between these objects. The transformation of the points against the simulation from (4) will look as follows:

(5) $$P_{iO}=T_{Sjref\to Rb}^{-1}\cdot T_{Rb\to O}^{-1}\cdot P_{i_{Sjref}}$$

where $P_{i_{Sjref}}$ is the *i*-th point expressed in the coordinate system of the *j*-th sensor, $T_{Sjref\to O}^{-1}$ is the transformation from the coordinate system of the *j*-th sensor to the coordinate system of the robot base, $T_{Rb\to O}^{-1}$ is the transformation from the robot base to the object coordinate system, and $P_{iO}$ is the i-th point expressed in the component coordinate system.

The results of the experiment are discussed in the next section.

## 4. Results and Discussion

**Figure 9:**
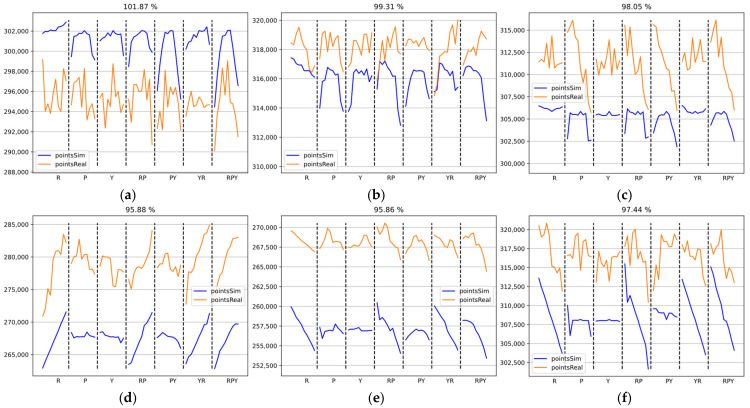

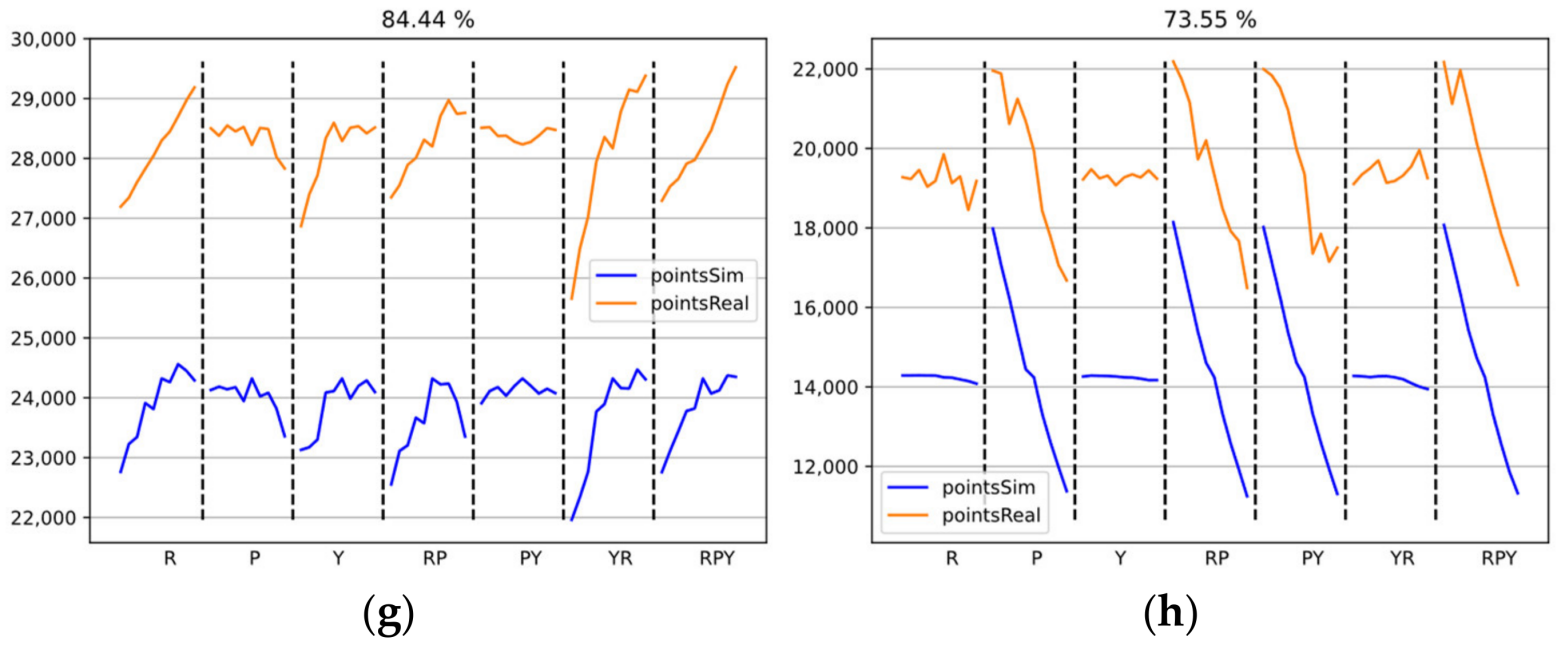
Comparison of the number of points in the point cloud obtained by each sensor during the whole measurement for Objects A, B, and C in the simulation (blue line) and on the real system (orange line). The Y-axis represents the number of points in the point cloud. The X-axis represents the measurement, where the individual rotation sequences are separated by a black dashed line (e.g., R is the rotation in Roll from −2° to +2°, RP is the rotation in Roll and Pitch, etc.). The percentages represent the match of the simulation with the actual measurement: (**a**) Object A, sensor no. 1; (**b**) Object A, sensor no. 2; (**c**) Object A, sensor no. 3; (**d**) Object B, sensor no. 1; (**e**) Object B, sensor no. 2; (**f**) Object B, sensor no. 3; (**g**) Object C, sensor no. 2; (**h**) Object C, sensor no. 3.

An indicator of the match between the simulation model and the real system is the number of points recorded during the measurement. Ideally, the number of points in the point cloud from the simulation should be identical to the point cloud from the real measurement. Figure 9a–h show the number of points in the point clouds collected by each sensor for all objects throughout the measurement. The X-axis represents the rotation of the object during the measurement; rotation around the axis is indicated by letters (R is rotation in Roll only; RP is rotation in Roll and Pitch at the same time; RPY is rotation in Roll, Pitch, and Yaw at the same time). In each section (separated by a black dashed line), measurements were taken while rotating the object from −2° to +2° in 0.4° increments. The Y-axis represents the number of points in the 3D point cloud, where the blue line represents the points from the simulation and the orange line represents the points from the real measurement.

Figure 9a–c shows the number of points in the point clouds for Component A, where Figure 9a shows data from Sensor S1, Figure 9b shows data from Sensor S2, and Figure 9c shows data from Sensor S3. For this object, the number of points in the point cloud is almost the same; the difference in the number of points between the actual 3D point cloud and the simulation point cloud was less than 2%.

Figure 9d–f represents the number of points in the point clouds for Component B. Figure 9d shows the data of Sensor S1, Figure 9e shows the data of Sensor S2, and Figure 9f shows the data of Sensor S3. For this object, the number of points captured in the simulation differed from the real measurement by less than 5%.

Figure 9g,h shows the number of points in the point clouds for Object C. We did not measure material properties for this object; since it is a very smooth surface, the material properties for the simulation were estimated as aluminum with a surface roughness of Ra0.8. For this object, the number of points captured in the simulation differed the most from the real measurement. Figure 9g represents the data of Sensor S2, where the number of points differed by almost 15.5%. Figure 9h represents the data of Sensor S3; the number of points differed by almost 26.5%. Sensor S1 data was not measured, since the simulation suggests that the best results are obtained by using only two sensors (S2 and S3).

The data collected from the real measurement were used as input to the object orientation calculation (see Algorithm 4), where the sensor configuration was selected according to the simulation results of Section 3.1. In the following graphs we can see a comparison of the calculated deviation (error) of the orientation according to the simulation (blue boxplot) and on the real system (orange boxplot).

**Figure 10:**
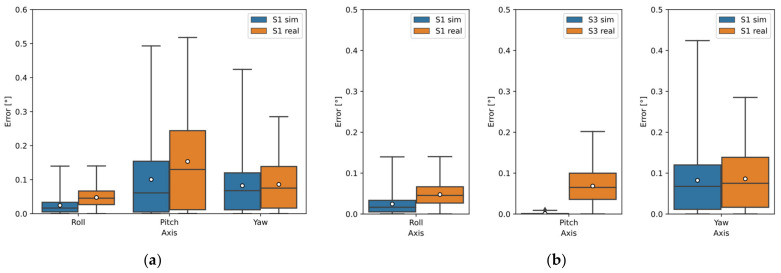

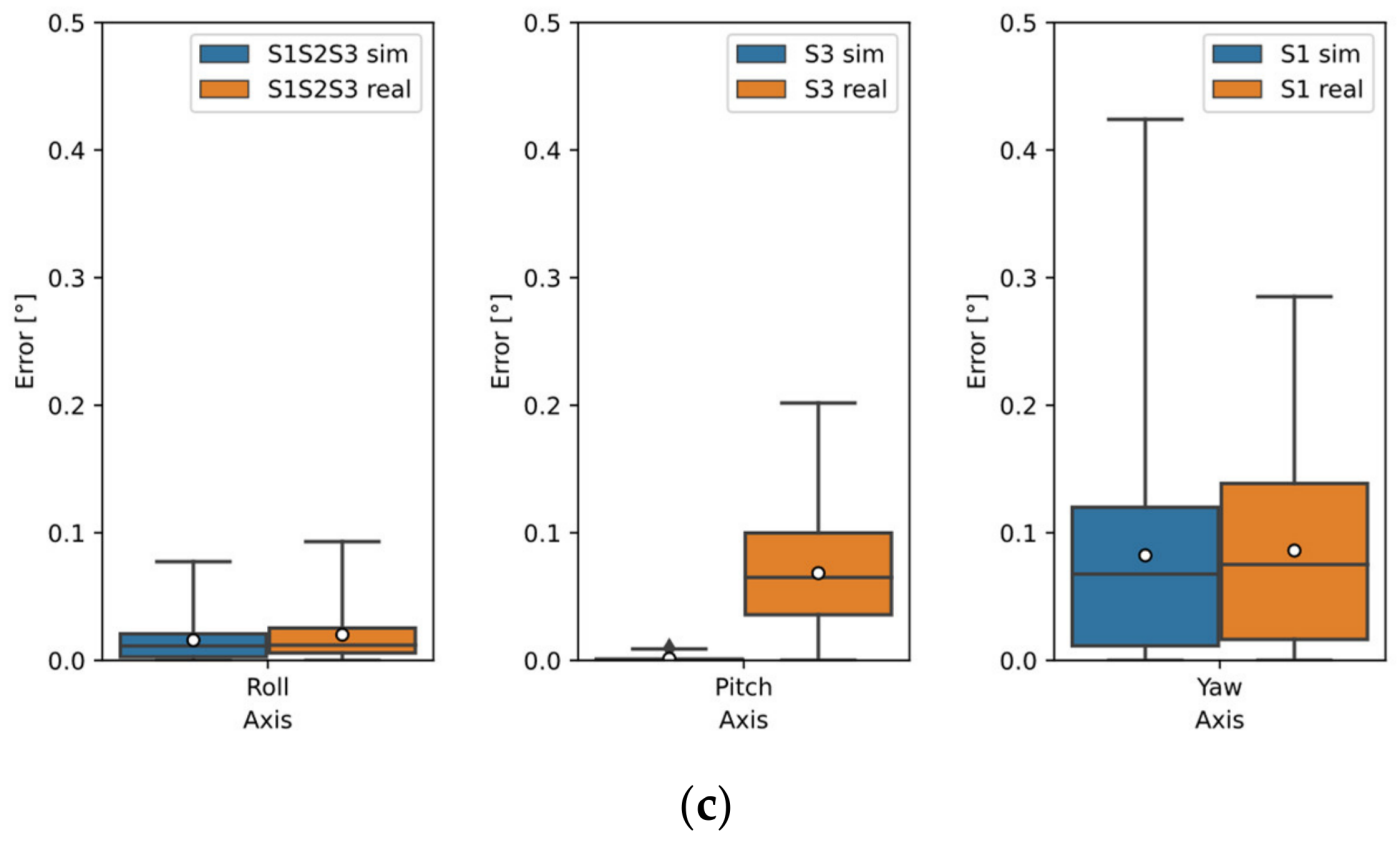
Comparison of results from simulation and real measurements of Object A: (**a**) using one sensor—S1; (**b**) configuration of two sensors: S1 and S3-S1 (Roll and Yaw detection) and S3 (Pitch detection); (**c**) configuration of three sensors—S1S2S3 (Roll detection), S3 (Pitch detection) and S1 (Yaw detection).

Figure 10a–c plots the deviations of the calculated orientation of Object A. The deviation using one sensor (S1) is shown in Figure 10a, where the median and range (75th percentile) of the Roll detection was below 0.075°; for the Pitch detection, the median error was below 0.15°, but the error range for the real sensor was higher (below 0.25°); for the Yaw detection, the median error was at 0.075° and the error range was below 0.15°.

The deviation using the two-sensor configuration can be seen in Figure 10b, where Sensor S1 was again used for the Roll and Yaw detection (so there is no improvement in these angles) and Sensor S3 was used for the Pitch detection, where the median error for the real measurement was around 0.08° and the range was at 0.1°, while for the simulation result these values are almost zero.

Using a three-sensor configuration, detection accuracy was only improved in Roll where all sensor data were an input into the calculation and the median error was below 0.025° and the error range was below 0.035° (see Figure 10c). For Pitch detection, Sensor S3 was again used and for Yaw, Sensor S1 was used.

**Figure 11:**
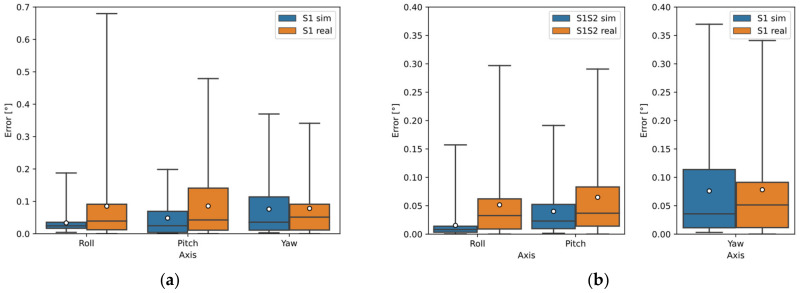

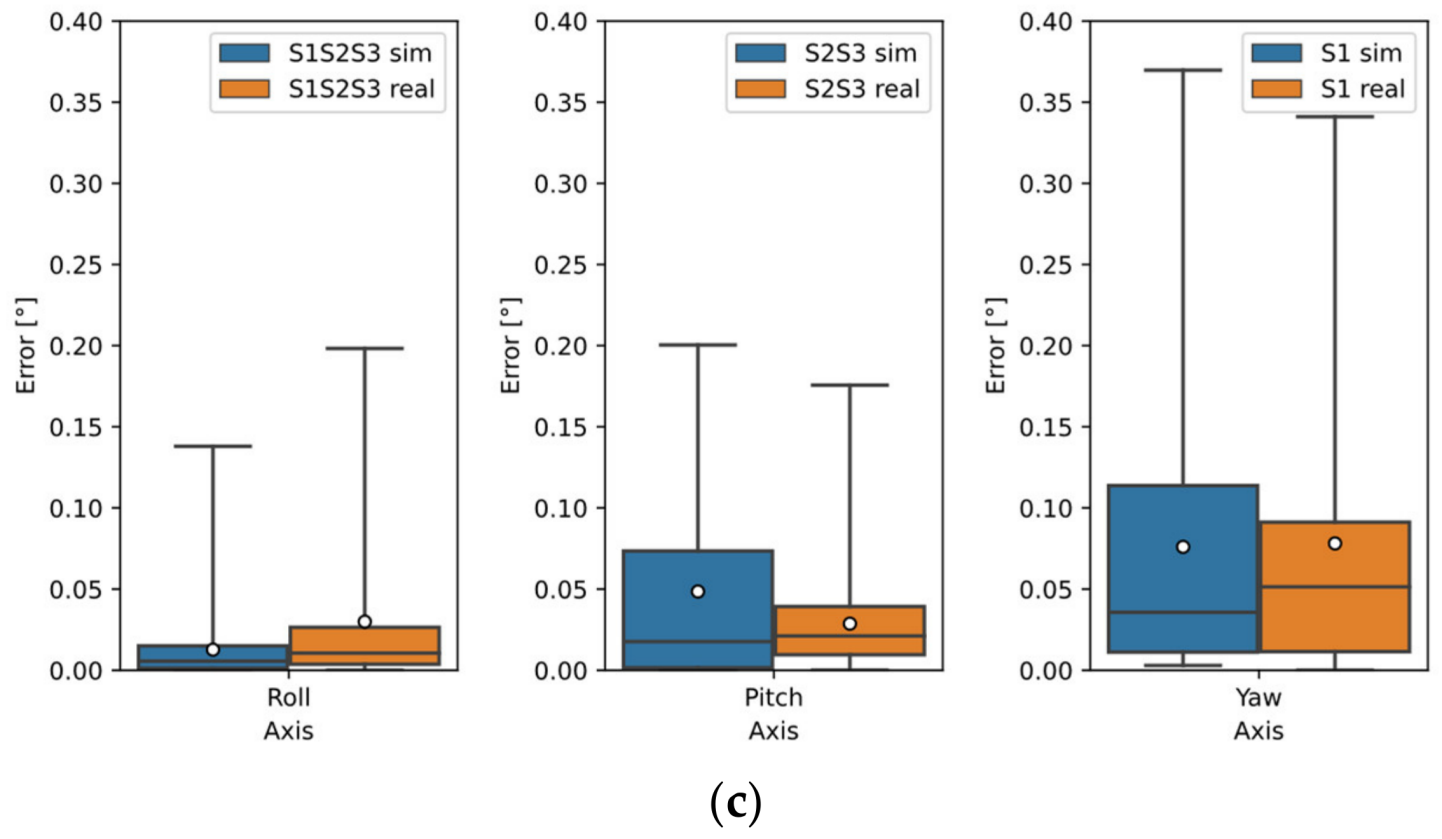
Comparison of results from simulation and real measurements of Object B: (**a**) using one sensor—S1; (**b**) configuration of two sensors: S1 and S2-S1S2 (Roll and Pitch detection), and S1 (Yaw detection); (**c**) configuration of three sensors—S1S2S3 (Roll detection), S2S3 (Pitch detection), and S1 (Yaw detection).

In Figure 11a–c, the deviations of the calculated orientation of Object B are plotted. The deviation using one sensor (S1) is shown in Figure 11a, where the median error for all angles was below 0.05°, the range of detection error in Roll and Yaw was below 0.1°, and the error range in Pitch was below 0.15°.

The deviation using the two-sensor configuration can be seen in Figure 11b, where the combined data from Sensors S1 and S2 were used for Roll and Pitch detection and Sensor S1 was used for Yaw detection. The median error at all angles was below 0.05° and the error range was below 0.1°.

Using a three-sensor configuration, the detection in Roll and Pitch was improved, with all sensor data input to the Roll calculation and S2 and S3 sensor data input to the Pitch calculation. The median error in Roll and Pitch was below 0.025° and the range was below 0.05°. For Yaw detection, again only Sensor S1 was used.

**Figure 12:**
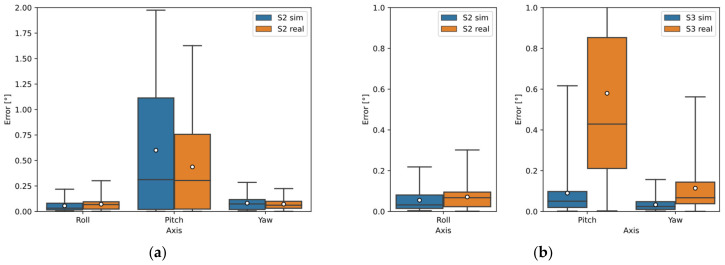
Comparison of results from simulation and real measurements of object C: (**a**) using one sensor—S2; (**b**) configuration of two sensors: S2 and S3-S2 (Roll detection) and S3 (Pitch and Yaw detection).

In Figure 12a,b, the deviations of the calculated orientation of Object C are plotted. The deviation using one sensor (S2) is shown in Figure 12a, where the median error for Roll and Yaw is approximately 0.06° and the error range is below 0.1°. The median error in Pitch is 0.3° and the error range is 0.75° for the real measurement.

The deviation using the two-sensor configuration can be seen in Figure 12b, where the S2 sensor was again used for Roll detection. Sensor S3 was used to detect Pitch and Yaw, where the median error in Pitch for the real measurement was slightly above 0.4° and the error range was closely above 0.8°; while for the simulation, the median and range were below 0.1°. For Yaw detection, the median error was 0.06° and the range was below 0.15°.

For Objects A and B, the simulation results were almost identical to the actual measurement results. The number of points in the 3D point clouds obtained by simulation and actual measurements differed by a maximum of 2% for Object A and a maximum of 5% for Object B. The results of the orientation estimation from the simulation data differed from the actual data by at most one tenth of a degree which is understandable because there are always some inaccuracies in a real system (manufacturing, assembly, etc.). Moreover, a deviation of a tenth of a degree is negligible, considering that the UR10e is a collaborative robot and is not as rigid and precise as industrial robots.

The simulation results for Object C were more different from the real ones. The number of points in the 3D point clouds from the simulation differed from the actual by 15.5% for Sensor S2 and up to 26.5% for Sensor S3. The results of the simulation orientation estimation differed from the actual results sometimes by up to 0.4° (median error) and the error range by up to 0.8° (75th percentile). Using one sensor for Pitch detection, the real system was more accurate than the simulation (the error range was 0.25° smaller for the real system, see Figure 12a). Whereas using a two-sensor configuration, where the S3 sensor data were used to calculate Pitch, the error for the real system was noticeably larger (according to the simulation, the error range was less than 0.1° and by the real measurement the error range was almost 0.9°). These differences in the simulation model were due to the assignment of incorrect material properties to object C in the simulation. The material of this object was assigned as an estimated smooth aluminum alloy with a surface roughness of Ra0.8 because the actual material properties (smooth steel) of object C were not available. In reality, the laser reflected more diffusely from this object than the assigned material in the simulation, hence the real sensor captured up more points, as shown in Figure 9g,h.

In case of Object A, these sensors achieved the best results:

- Using one sensor—Sensor S1;
- Using two sensors—Sensors S1 (for Roll and Yaw detection) and S3 (for Pitch detection);
- Using three sensors—Sensors S1S2S3 (for Roll detection), S3 (for Pitch detection) and S1 (for Yaw detection).

For Object B, the best results were achieved by:

- Using one sensor—Sensor S1;
- Using two sensors—S1S2 (for Roll and Pitch detection) and S1 (for Yaw detection);
- Using three sensors—Sensors S1S2S3 (for Roll detection), Sensors S2S3 (for Pitch detection) and S1 (for Yaw detection).

For Object C, the best results were achieved by:

- Using one sensor—Sensor S2;
- Using two sensors—S2 (for Roll detection) and S3 (for Pitch and Yaw detection);
- Using three sensors—the three-sensor configuration did not achieve better results than the two-sensor configuration.

The presented results show that the pose of the sensors with respect to the scanned object has an important impact on the accuracy of the orientation estimation. The results show that the data from one correctly placed sensor could be more relevant than the data from more sensors for object pose estimation, thus the number of sensors can be minimized by appropriate placement of sensors.

Table 1 provides an overview of the selected configurations based on the simulation (see Section 3.1) and a comparison of the simulation results with the real measurements of all objects. One has to choose which configuration will be the most suitable for his task; it depends on the required detection accuracy and when it is worth investing in more sensors.

## 5. Conclusions

Tightening up tolerances in industrial manufacturing and assembly is essential to improve product quality and durability. One of the reasons for tightening is less stress on parts during precision assembly. However, precision assembly is more time-consuming, as parts must be precisely positioned at the pick points so a machine (mainly an industrial robot) can accurately grasp them. To speed up the process, bin-picking systems are used to estimate the pose of the object in a bin. However, the accuracy of pose estimation is about ±2°. Objects grasped in this way must be aligned to the exact position using other devices or the position of the grasped object in the robot’s end-effector must be estimated.

In this paper, we have investigated the methodology for selecting the configuration of LLT sensors relative to the scanned object based on simulation. When the sensors are correctly placed, relevant data are collected for the object orientation estimation algorithm, thus improving the orientation estimation.

For our experiment we selected three objects. Each object had a different shape and material and was made with a different technology. Object A was made of aluminum by milling, Object B was made of PLA plastic by additive technology (3D printing), and object C was made by bending a steel cut-out.

Based on the virtual scanning, a dataset was prepared to estimate the orientation of objects in 3D space. For each object, the sensor configurations with the smallest orientation estimation error were selected using one, two, and three sensors. The simulation results were verified using actual measurements. To place the LLT sensors in 3D space, housings were made using 3D printing and a UR10e robot was used to rotate and move the objects.

A comparison of the simulated and actual measurements is shown in Table 1, which shows the detection error (median) and the error range (75th percentile) for all objects and selected sensor configurations.

The presented data show that the position of the sensors relative to the scanned object has a significant effect on the accuracy of the orientation estimation. The results show that the number of sensors can be minimized by appropriate placement of sensors. Using input data from all three sensors may not always lead to improved orientation estimation, but input data from one sensor may show better results. When the correct optical properties of the scanned object are set, the simulation matches the actual measurement. Thanks to the virtual environment, we can find the optimal position of the sensors and improve the orientation estimation system. This also allows working with LLT sensors in the simulation, where the user can test their scanning behavior before acquiring the real sensors.

Now, it is necessary to place sensors to each object manually, based on the user’s decision. This opens up the possibility for future work by creating an algorithm that automatically selects the optimal sensor placement for a given object of manipulation.

## Figures and Tables

**Table 1:** Detection error (median and 75th percentile) of selected configurations based on simulation and comparison of measurement results on the real system for all objects. All values are in degrees.

Object	Number of Sensors	Sensor Configuration	Simulation Error (Median)	Actual Error (Median)	Simulation 75th Percentile	Actual 75th Percentile
A	1	Roll—S1	0.016	0.045	0.033	0.066
		Pitch—S1	0.061	0.130	0.154	0.243
		Yaw—S1	0.067	0.075	0.120	0.138
	2	Roll—S1	0.016	0.045	0.033	0.066
		Pitch—S3	0.004	0.065	0.009	0.099
		Yaw—S1	0.067	0.075	0.120	0.138
	3	Roll—S1S2S3	0.011	0.012	0.020	0.025
		Pitch—S3	0.0004	0.065	0.009	0.099
		Yaw—S1	0.067	0.075	0.120	0.138
B	1	Roll—S1	0.024	0.039	0.035	0.091
		Pitch—S1	0.024	0.042	0.069	0.140
		Yaw—S1	0.035	0.051	0.113	0.091
	2	Roll—S1S2	0.008	0.032	0.013	0.062
		Pitch—S1S2	0.023	0.036	0.052	0.083
		Yaw—S1	0.035	0.051	0.113	0.091
	3	Roll—S1S2S3	0.005	0.010	0.014	0.026
		Pitch—S2S3	0.017	0.021	0.073	0.039
		Yaw—S1	0.035	0.051	0.113	0.091
C	1	Roll—S2	0.032	0.067	0.080	0.094
		Pitch—S2	0.312	0.304	1.113	0.756
		Yaw—S2	0.073	0.061	0.116	0.099
	2	Roll—S2	0.032	0.067	0.080	0.094
		Pitch—S3	0.050	0.428	0.097	0.852
		Yaw—S3	0.024	0.067	0.048	0.143

## Data Availability

Not applicable.

## Abbreviations

The following abbreviations are used in this manuscript:

RANSAC	RANdom SAmple Consensus
ICP	Iterative Closest Point
AoI	Angle of Incidence
LLT	Laser Line Triangulation
CCD	Charge Coupled Device
